# Non-Thermal Biocompatible Plasma Jet Induction of Apoptosis in Brain Cancer Cells

**DOI:** 10.3390/cells10020236

**Published:** 2021-01-26

**Authors:** Mahmuda Akter, Jun Sup Lim, Eun Ha Choi, Ihn Han

**Affiliations:** 1 Department of Plasma Bio-Display, Kwangwoon University, Seoul 01897, Korea; nipa21stfeb@gmail.com; 2 Plasma Bioscience Research Center, Applied Plasma Medicine Center, Kwangwoon University, Seoul 01897, Korea; junsup117@nate.com; 3 Department of Electrical and Biological Physics, Kwangwoon University, Seoul 01897, Korea

**Keywords:** non-thermal biocompatible plasma, NBP, glioblastoma multiforme, U87 MG, apoptosis pathway, p53, MAPK pathway, p38, ERK, JNK

## Abstract

Glioblastoma multiforme (GBM) is a highly malignant and rapidly advancing astrocytic brain tumor in adults. Current therapy possibilities are chemotherapy, surgical resection, and radiation. The complexity of drug release through the blood-brain barrier, tumor reaction to chemotherapy, and the inherent resistance of tumor cells present challenges. New therapies are needed for individual use or combination with conventional methods for more effective treatment and improved survival for patients. GBM is difficult to treat because it grows quickly, spreads finger-shaped tentacles, and creates an irregular margin of normal tissue surrounding the tumor. Non-thermal biocompatible plasma (NBP) has recently been shown to selectively target cancer cells with minimal effects on regular cells, acting by generating reactive oxygen species (ROS) and reactive nitrogen species (RNS). We applied a soft jet plasma device with a syringe shape to U87 MG cells and astrocytes. Our results show that NBP-J significantly inhibits cell proliferation and changes morphology, induces cell cycle arrest, inhibits the survival pathway, and induces apoptosis. Our results indicate that NBP-J may be an efficient and safe clinical device for brain cancer therapy.

## 1. Introduction

Brain cancer is one of the aggressive cancers in humans and is a serious health and social concern. Of all human brain cancers, GBM is the most recurrent brain cancer, with a mean survival rate of 12–15 months [1,2,3]. A combination of chemotherapy, radiotherapy, and surgical resection is the gold standard for glioblastoma treatment. However, with conventional cancer treatment methods, glioblastoma can cause strong resistance. Due to limited therapy options and poor diagnosis, glioblastoma has received increased attention from researchers who seek to develop novel therapies and successful selective cancer treatments. Glioblastoma tumors predominantly originate deep in the brain. New therapy options are needed to treat the cancer and to reduce damage to normal tissues. For cancer-selective treatment, Feil et al. [4] reported the effects of a non-thermally operated Martin Argon Plasma Beamer System (MABS) on cervical cancer cell lines and non-cancerous cervical tissue cells. Weiss et al. [5] presented an electron-spin resonance spectroscopy on human tissue samples and treated with different cold atmospheric plasma (CAP) doses, allowing the distribution and the measurement of consequential radicals in the tissues. The plasma-dependent fluctuations of different molecular components of solid human cervical epithelium and cervical cancer cells are determined by Wenzel et al. [6].

Non-thermal atmospheric pressure biocompatible plasma (NBP) is an emerging novel therapy to selectively kill cancer cells [7,8]. Plasma is an ionized gas containing free charges, free radicals, neutral atoms, and photons (UV)—that is, a gaseous matter with quasi-neutral charge [9,10,11]. Studies have established the effectiveness of sterilization by NBP [12,13,14,15,16]. Furthermore, recent studies have shown that NBP applications can selectively induce apoptosis in different cancer cell lines in vitro [17,18,19,20]. The synergistic purpose of cold atmospheric plasma with different approaches, for example, nanotechnologies, pulsed electric field, and extra static magnetic field, will improve the efficiency of CAP treatment [21,22,23,24,25,26]. Non-thermal plasma may temporarily increase the permeability of the cell membrane to increase endocytosis to absorb the gold nanoparticles, resulting in synergistic cytotoxicity to the target cancer cells, as presented by He et al. [23]. Chen et al. [24] research group has tested CAP on glioblastoma tumors in mice while Fe^2+^/Fe^3+^ from iron oxide-based magnetic nanoparticles can transform H_2_O_2_ into **•**OH by Fenton’s reaction. Most generally, in vitro analysis has shown a link or correlation between plasma dose and observed cell effects [27]. Xiaoqian et al. reported that ROS are supposed to play significant roles in cell lipid peroxidation and other effects. Plasma has revealed the least effect on neighboring (healthy) tissue, via permitting competent biological action within a minute. Plasma generates reactive oxygen and nitrogen species that can successfully kill several types of cancer cells [28,29,30,31]. NBP is a cocktail containing different reactive oxygen and nitrogen species (RONS), neutral particles, other charged particles, and electrons. Reactive ionized species, including H_2_O_2_, OH∙, O_2_∙^−^, N_2_^+^, and NO, are key components of the NBP-J that possess therapeutic properties, not only with regard to the tumor, but also in disinfection of biological agents [32], destruction of viruses [33], and healing of wounds [34,35]. ROS are known to moderate oxidative stress, play a role in cellular damage, and activate precise cell death pathways. ROS can trigger cell signaling cascades, including those connecting several diverse mitogen-activated protein kinase (MAPK) cascades [35,36]. Plasma treatment has revealed powerful and selective anti-cancer capabilities in different cancer cell lines, including colorectal, breast, skin, cervical, and brain cancers [37].

In our previous study [36], we showed that NBP-J can be applied to brain tumors. In this study, we used NBP-J in the presence of an air-gas flow to assess the effects of plasma on cell death in glioblastoma. We investigated the potential of plasma for brain cancer cells to alter the oxidative stress pathway and identified PI3K/Akt, P53 and total MAPK signaling pathways as part of the anti-cancer effects of NBP-J.

## 2. Materials and Methods

### 2.1. Cell Culture

U87 MG and U251 (human brain glioblastoma cancer cell line) and HCT-116 (human colon cancer cell line) were obtained from the Korean Cell Line Bank (KCLB, Seoul, Korea), and normal human astrocytes were obtained from Lonza (CC-2565, Basel, Switzerland). U87 MG, U251, and astrocyte cells were cultured in Dulbecco’s modified Eagle’s medium (DMEM) supplemented with 10% fetal bovine serum (FBS, Gibco, NY, USA) and 1% antibiotics (Life Technologies, Gibco, Grand Island, NY, USA). HCT-116 cell lines were cultured in Roswell Park Memorial Institute (RPMI) 1640 medium supplemented with 10% FBS and 1% antibiotics. Suspensions of cells were cultured in 100 mm cell culture dishes (Corning, New York, NY, USA) and incubated for approximately 20 to 24 h to reach confluency for experiments. Cell cultures were maintained in a humidified incubator containing 5% CO_2_ at 37 °C. Cells were observed under a Nikon Eclipse TS100 inverted microscope.

### 2.2. Plasma Device Configuration and Non-Thermal Biocompatible Plasma Jet (NBP-J) Treatment

**Figure 1:**
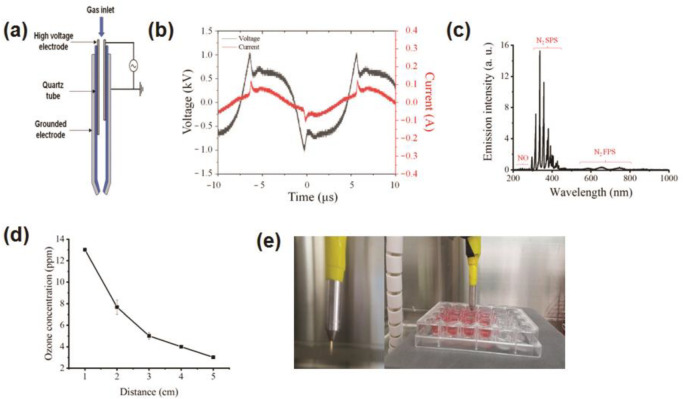
(**a**) Schematic outline of the experimental setup. (**b**) Current and voltage waveforms through plasma discharge. (**c**) Optical emission spectra (OES) measurement for soft plasma jet device by air gas. (**d**) Measurement of ozone concentration at different distances of treatment. (**e**) Photograph of the soft plasma jet.

Figure 1a shows the schematic structure of the NBP-J used in this study. Atmospheric air was used to generate plasma, which consisted of two electrodes, a high-voltage power supply, and dielectrics. The feed air gas flow was controlled by an analog controller. A voltage controller was used to regulate the primary voltage. The inner electrode is a typical injection needle composed of stainless steel, 1.20 mm × 0.27 mm. The outer electrode is also composed of stainless steel. The thickness of the outer electrode was 0.27 mm with a length of 6 mm and a centrally perforated hole of 0.70 mm to generate plasma. The discharge gap distance between the inner and outer electrodes was 2 mm. A GWINSTEK oscilloscope with a high-voltage probe (P6015A, Tektronix, Beaverton, OR, USA) and a current probe (P6021A, Tektronix) were used to measure the voltages and currents, respectively.

Generation of reactive oxygen or nitrogen species produced from the plasma was confirmed by optical emission spectroscopy (HR4000, Ocean Optics, Dunedin, FL, USA). An ozone meter (SKT-9300, 0.1 to 100 ppm, resolution 0.1 ppm) was used to determine the concentration of ozone. The gap distance to the media surface from the tip of the plasma jet was set to 5 mm in this experiment.

### 2.3. Cell Viability (Alamar blue) Assay

Cells were seeded into 24-well plates (Falcon, BD Biosciences, San Jose, CA, USA) at a concentration of 1 × 10^5^ cells/well and incubated for 24 h at 37 °C in 5% CO_2_ atmosphere. Cells were treated with NBP-J for 10, 30, 60, and 180 s, followed by an additional incubation. Cell viability was evaluated using the Alamar blue assay, a redox fluorogenic symbol of metabolic reduction (Fischer Scientific, Ballycoolin, Ireland). The control group without plasma treatment was evaluated by all assays. All assays were performed using three independent sets of tests.

### 2.4. Intracellular ROS Detection

U87 MG cells were seeded at 5 × 10^4^ on round glass coverslips in 12-well plates and incubated for 24 h at 37 °C in a 5% CO_2_ atmosphere. Afterward, cells were treated with NBP-J at different treatment times (10, 30, 60, and 180 s). 2′,7′ Dichlorodihydrofluorescein diacetate (H_2_DCFDA, Invitrogen, CA, USA) was purchased for intracellular ROS measurement according to the manufacturer’s protocol. Fluorescence images were obtained by laser scanning confocal microscopy (Zeiss, LSM 510, Little Rock, AR, USA) at 40× magnification. Experimental procedures were performed according to the manufacturer’s instructions.

### 2.5. Cell Cycle Analysis by Flow Cytometry

For cell cycle analysis of the U87 MG, cell lines were seeded at 4 × 10^5^ cells per well and incubated for 24 h. At each time point, cells were trypsinized, collected, and set with ice-cold 75% ethanol for 24 h. Cells were washed twice with PBS and stained with propidium iodide (PI) and RNase-2 (Sigma-Aldrich, St. Louis, MO, USA). Stained cells were then analyzed by flow cytometry.

### 2.6. Immunofluorescence Staining

For immunofluorescence staining of p-Akt (Cell Signaling, 1:1000, Danvers, MA, USA) after NBP-J treatment, U87 MG cells were fixed with 4% paraformaldehyde solution for 20 min. After rinsing with PBS solution, cells were permeabilized, and the blocking procedure was sequentially completed using PBS containing 0.25% Triton X-100 (Fisher Scientific) and 10% BSA in TBS with 0.1% Tween 20 for 1 h. Cells were then incubated at 4 °C overnight with the primary antibody in a humidified chamber. Cells were washed three times with PBS solution and then incubated in goat polyclonal secondary antibody to mouse IgG-H&L (Alexa Fluor 594, BioRad, 1:5000, CA, USA) for 60 min in the dark. To identify the nuclei, cells were stained with Hoechst 33,258 for 15 min. Samples were analyzed using a confocal laser scanning fluorescence microscope.

### 2.7. Western Blot Analysis

After NBP-J treatment, cells were lysed with RIPA buffer for Western blotting. Proteins were separated by 4–15% SDS-PAGE (sodium dodecyl sulfate-polyacrylamide gel electrophoresis) (Bio-Rad, Hercules, CA, USA) and transferred to a nitrocellulose membrane (Millipore, Billerica, MA, USA). The membrane was blocked with 0.5% bovine serum albumin (BSA) using SNAP i.d^®^ 2.0 (Darmstadt, Germany) at room temperature and incubated with primary antibodies specific to p53, caspase 3, caspase 7, PARP, MAPK, PI3K, p-Akt (Cell Signaling, Danvers, MA, USA), and β-actin. Subsequently, anti-mouse and anti-rabbit HRP (horseradish peroxidase)-conjugated secondary antibodies (Serotec, Kidlington, UK) were used to detect the primaries. Band intensity was detected and analyzed by enhanced chemiluminescence (ChemiDocTM System, Bio-Rad).

### 2.8. Statistical Analysis

All values are presented as means and standard deviations (S.D.) of at least three independent sets of experiments. Student’s t-tests were used to evaluate statistical significance (* *p* < 0.05, ** *p* < 0.01, and *** *p* < 0.001 indicate significance).

## 3. Results

### 3.1. Soft Jet Plasma and Its Physical Characterization

Figure 1a illustrates the schematic of the soft jet plasma setup used in this study for measuring optical emission spectra, current, voltage, and other parameters. The airflow rate was set at 1 lpm for the operation system. The gap between the discharge of the plasma tip and the surface of the media was fixed at 5 mm. Figure 1b shows typical current and voltage waveforms of the discharge using air. Here, the peak voltage and current are shown to be approximately 1 kV and 100 mA, respectively. Figure 1c shows optical emission spectra of soft jet plasma from 200 to 1000 nm in this experiment. The spectra of the soft jet plasma consist mainly of N_2_ nitrogen second positive system (N_2_ SPS) ranging from 311 to 380 nm, corresponding to the transition ($N_{2}\left(C^{3}\Pi_{g}-B^{3}\Pi_{g}\right)$). However, in this plasma, NO_Y_ ranging from 210 to 280 nm and N_2_ nitrogen first positive system (N_2_ FPS) ranging from 560 to 800 nm, corresponding to the transition ($N_{2}\left(B^{3}\Pi_{g}-A^{3}\Sigma_{u}^{+}\right)$), were also detected. These excited nitrogen species are also reactive species that can oxidize several biological processes by taking electrons from them. Air-gas was used as the working gas for the biological experiments. NBP-J also produces a significant quantity of ozone (O_3_), which is known to have a strong destructive effect on cells. Figure 1d shows the ozone produced by treated plasma from different distances. Ozone concentration reached 13.03 ppm when the distance was 1 cm from the tip of the plasma source and decreased to 7.68, 5.01, 4.0, and 3.02 ppm with distances of 2, 3, 4, and 5 cm, respectively. Figure 1e shows the experimental soft jet plasma used in this study.

### 3.2. Cell Viability Assay

**Figure 2:**
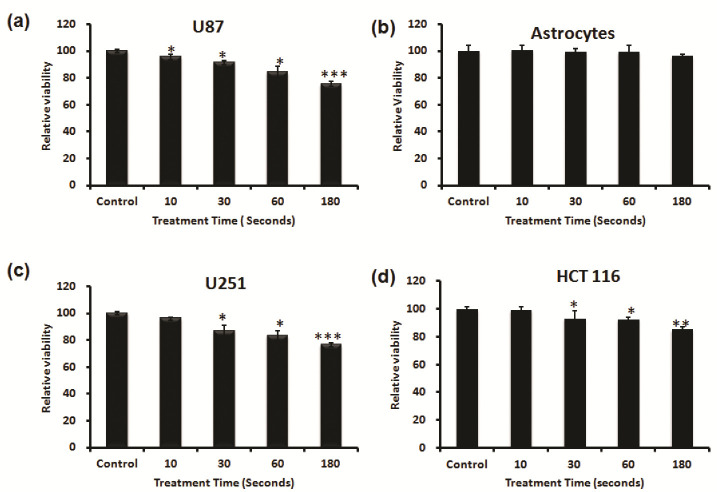
Effects of NBP-J on cell viability of (**a**) U87 MG, (**b**) astrocytes, (**c**) U251, and (**d**) HCT116 cell lines for different treatment durations from 10 to 180 s. An Alamar blue assay was performed after 24 h of treatment. Data are presented as means ± SD of three independent sets of experiments. * *p* < 0.05, ** *p* < 0.01, and *** *p* < 0.001.

First, we investigated the capacity of NBP-J to selectively kill cancer cells. We selected three cancer cell lines (U87 MG, U251, HCT116) and one normal astrocyte cell line. Both cancer and normal cells were treated for different durations from 10 to 180 s to check the cell viability after NBP-J treatment. As shown in Figure 2a, the viability of U87MG cells decreased significantly in a time-dependent manner after NBP-J treatment. Figure 2b shows that NBP-J treatment from 10 to 180 s did not induce any significant decrease when compared to the untreated group for astrocyte cell viability. Figure 2c,d shows treated U251 and HCT116 cells with NBP-J after 24 h of seeding, along with a non-treated group that decreased significantly compared to control.

**Figure 3:**
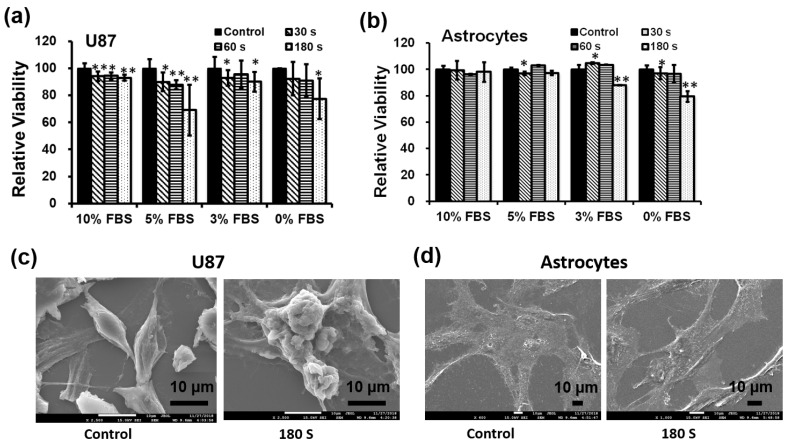
(**a**) Effects of NBP-J on cell viability of U87 MG cell line and (**b**) Astrocytes treated with different concentrations of FBS (fetal bovine serum) for various durations. An Alamar blue assay was performed after 24 h of treatment. Changes in morphology after plasma treatment in (**c**) U87MG and (**d**) astrocytes were viewed by SEM. Data are presented as means ± SD of three independent sets of experiments. * *p* < 0.05, ** *p* < 0.01, and *** *p* < 0.001.

Second, we investigated what percentage of fetal bovine serum (FBS) was able to affect cancer cells. We detected the cytotoxicity of only U87 MG cells and astrocytes using different percentages of FBS media. At the time of treatment, 10%, 5%, 3%, and 0% FBS with DMEM media was used. Cells were treated with NBP-J for various treatment times, including 30, 60, and 180 s, with a non-treated group as the control. Figure 3a shows that after 24 h of incubation, U87 MG cells treated with NBP-J showed decreased cell viability by approximately 5%, 30%, 10%, and 20% after 180 s of treatment with 10%, 5%, 3%, and 0% FBS, respectively. Figure 3b shows that in astrocytes treated with NBP-J and 5% or 10% FBS, cell viability was not significantly decreased, but in 3% and 0%, FBS viability was decreased. The percent of viable cells was reduced in the NBP-J-treated U87MG cells in a time-dependent manner. Finally, we selected 10% FBS to minimize astrocyte effects for the rest of our experiments.

### 3.3. Morphological Characteristics after Plasma Treatment

To examine the cell death type caused by NBP-J, microscopic observation was performed to observe cell morphology. Figure 3c shows the remarkable differences of the NBP-J-treated U87 MG cells at 180 s compared with the control. For astrocytes in Figure 3d, the morphology shows almost the same growth pattern after NBP-J treatment. After NBP-J treatment, cancer cells isolated from the surface, and numerous cells progress from a spindle shape to a cobblestone-like morphology. Morphology study revealed that the development pattern of the U87 MG cells was affected by NBP-J treatment, but the same was not true for astrocytes. There was a noticeable difference in the development pattern of treated U87MG cells compared to the untreated control. Morphological changes in NBP-J-treated U87 MG cells may indicate apoptotic body-like cells. However, in the case of astrocytes after NBP-J treatment, intact morphology is apparent. Overall, NBP-J treatment can inhibit the growth pattern of U87 MG cells but not of astrocytes.

### 3.4. Intracellular ROS Are Induced by Soft Plasma Jet

**Figure 4:**
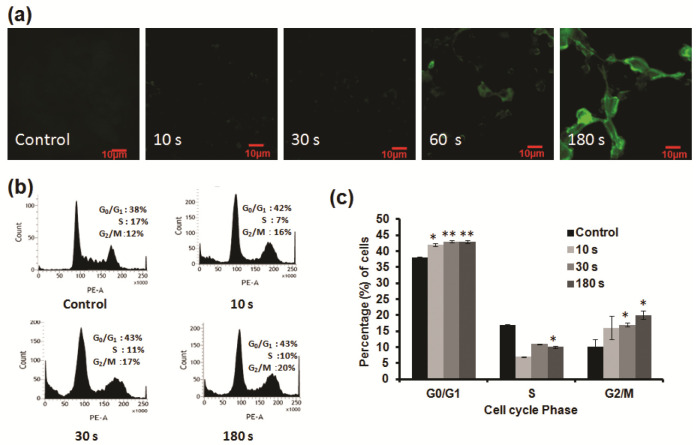
(**a**) Measurement of intracellular ROS levels in U87 MG cells by NBP-J using confocal microscopy. (**b**) Cellular responses in U87 MG cells after NBP-J treatment showing the cell cycle phase distribution. (**c**) Bar graph summarizing the percentage of cells in different cell cycle phases. Cells were analyzed by flow cytometry. Data are presented as means ± SD of three independent sets of experiments. * *p* < 0.05, ** *p* < 0.01.

NBP can generate ROS, thereby directly inducing apoptosis of cancer cells. As seen in Figure 4a, higher levels of ROS fluorescence were detected after subsequent NBP-J treatment in U87 MG cells compared to untreated controls. To evaluate cytotoxicity from ROS generated by NBP, U87 cells were preloaded with the cell-permeable ROS-sensitive fluorescent dye H_2_DCFDA. ROS generation was first visualized by confocal microscopy, confirming that NBP induced ROS-dependent H_2_DCFDA fluorescence in cell lines. The level of fluorescence in U87 MG cells seemed to be higher, in a time-dependent manner, than in untreated cells. These data specify that the longer NBP-J treatment, the greater the quantity of ROS that cells formed. NBP-J can generate ROS and, as a result, the created ROS might specifically relate to the induction of apoptosis.

### 3.5. NBP-J Induced G_0_/G_1_ Phase Cell Cycle Arrest of U87 MG Cells

We investigated the analysis of U87 MG cell cycle to elucidate the effects of NBP-J. Figure 4b,c shows the difference in the percentage of cells in G_0_/G_1_ phase. The percentage of cells in the G_0_/G_1_ phase was more noticeable than in the other phases, compared to the control. However, most cells started to accumulate at the G_0_/G_1_ phase and later accumulated at the G_2_/M phase after NBP-J treatment. The S phase population also represents apoptotic cells, although it did not increase the cell population. We hypothesized that plasma can induce the arrest of early G_0_/G_1_ phase, which is also linked with the alteration of proteins that play a role in cell cycle regulation. Based on our findings, we assumed that NBP-J influences cell cycle regulation.

### 3.6. NBP-J Treatment Induces Apoptosis through the MAPK and PI3K/Akt Pathways

**Figure 5:**
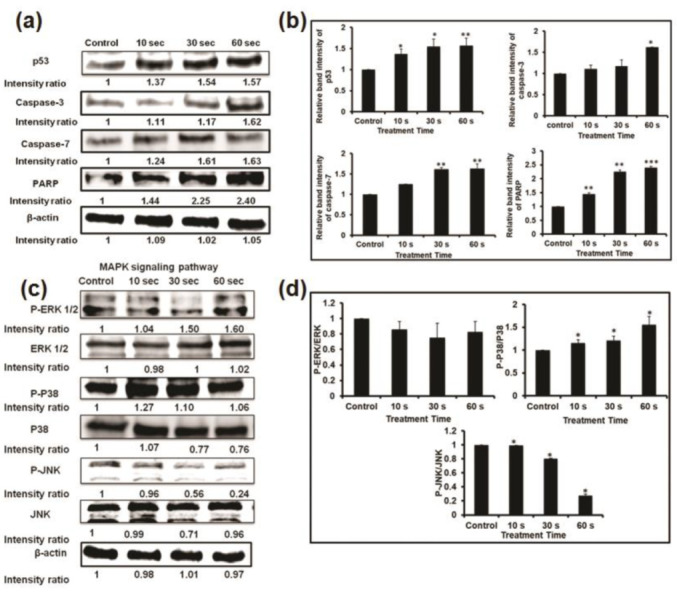
(**a**) Analysis of Western blot in U87MG cells for apoptosis-related protein expression. (**b**) Bar graph summarizing the apoptotic protein levels by relative band intensity. (**c**) Western blot analysis of the MAPK signaling pathway involved in the inhibition of the phosphorylation of ERK, p38, and JNK MAPKs by NBP-J treatment in U87MG cells. (**d**) Bar graph summarizing the phosphorylation of MAPKs. Data are presented as means ± SD of three independent sets of experiments. * *p* < 0.05, ** *p* < 0.01, and *** *p* < 0.001.

To investigate the mechanisms of apoptosis in U87 MG cells induced by NBP-J, apoptosis regulatory proteins (p53, caspase-3, caspase-7, and PARP) were detected by Western blotting, as shown in Figure 5a. p53 is one of the key tumor suppressors in response to genotoxic stresses. We analyzed p53 protein that showed an increasing pattern. Caspase-3, caspase-7, and PARP play vital roles in the apoptotic cellular population. Our results reveal that with the increase in plasma doses, the expression of apoptosis-related genes, including caspase-3, caspase-7, and PARP, increases (Figure 5a,b). The results of our study show that, following NBP-J treatment in U87 MG cells, caspase-3, caspase-7, and PARP proteins are activated, resulting in the activation of complex intracellular apoptotic cascades, and ultimately, promoting apoptosis.

**Figure 6:**
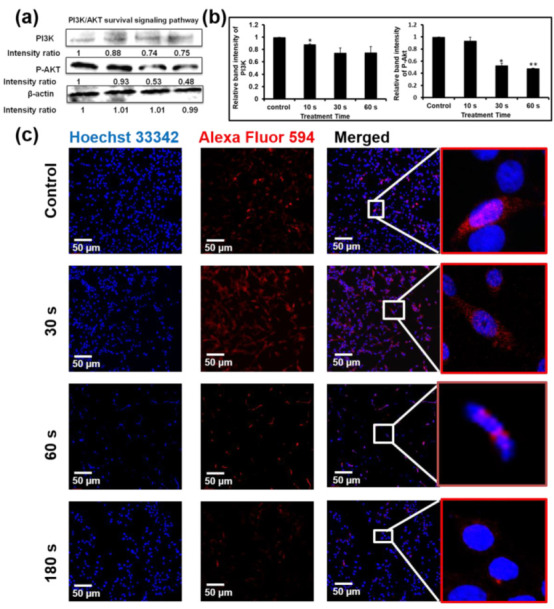
(**a**) Levels of PI3K and p-Akt were determined by Western blotting. (**b**) Bar graph representing the expression levels of PI3K and p-Akt. (**c**) Immunofluorescence-based visualization of p-Akt expression. Red: Fluorescence signal indicating marker expression. Blue: Nuclei visualized by Hoechst 33,342 dye. Confocal microscopy. 10× magnification. Scale bar = 100 µm. Data are presented as means ± SD of three independent sets of experiments. * *p* < 0.05, ** *p* < 0.01.

The effect of NBP-J on the MAPK and PI3K/Akt pathways was also examined and assayed by immunoblotting. Figure 5c,d indicates that NBP-J treatment decreased the phosphorylation of ERK1/2 and JNK, but increased the phosphorylation of p38 MAPK in an exposure time-dependent manner, although it did not decrease total expression levels. These results suggest that plasma-induced apoptosis occurs via regulation of the MAPK pathway. Western blotting in Figure 6a,b indicates that PI3K was lower in the plasma-treated group than in the control. In addition, the plasma treatment group exhibited decreased Akt phosphorylation. These results suggest plasma-induced apoptosis via inhibition of the PI3K/Akt pathway. β-actin was used as a loading control.

### 3.7. NBP-J Treatment Regulates the Possible Mechanism of Akt Signaling Pathway

Akt protein plays an important role in regulating a number of cellular processes, such as metabolism, growth, and survival. To further confirm apoptosis by the Akt pathway, we performed immunofluorescence staining. Figure 6c shows that after plasma treatment, phosphorylation of Akt gradually decreased compared to the control. p-Akt appeared as red fluorescence, while nuclei appeared (Hoechst 33,342 stain). Though our data show that p-Akt expression was not completely eliminated, levels were low after plasma treatment, indicating that this signaling pathway is affected by NBP-J treatment.

## 4. Discussion

Recently, non-thermal plasma has been increasingly considered for its prospective biomedical applications. Application of plasma has been used in the rising fields of plasma medicine, which includes microorganism inactivation, coagulation of blood, tooth bleaching, regeneration of skin, cancer therapy, and others. Effective treatment of different tumor cells in vitro and in vivo using plasma has been demonstrated [38]. Plasma contains reactive species that combine through cells. No negative effect on normal cells has been reported when optimal doses are used for cancer cell apoptosis [38,39,40]. However, in this study, we used NBP-J to treat brain cancer cells in vitro, with astrocytes as a control.

NBP-J-treated cancer cells undergo morphological changes and apoptotic induction, whereas ordinary cells are left comparatively unharmed. NBP-J resulted in a larger decrease in cell viability in the three selected cancer cell lines (U87 MG, U251, and HCT116). The cytotoxicity assay revealed that NBP has a killing effect on brain cancer cells, whereas cytotoxic effects were enhanced with the increased treatment time in different percentage of FBS. FBS is one of the most important supplements to culture the cells and it may have some serum effect during plasma treatment. NBP-J produces reactive species, which may affect the mechanism of cancer therapy [41]. Cancer cells are more vulnerable than normal cells, owing to their increased rates of ROS generation anywhere that support cancer cell proliferation and metastasis [42].

One of the major regulatory mechanisms of cell growth is cell cycle control [33,34,35,36,43,44,45,46]. Several anticancer agents have been published to induce apoptotic cell death to arrest the cell cycle at a particular checkpoint [43,45]. In this study, ROS uptake and cell percentage in G_0_/G_1_ phase increased in a time-dependent manner by NBP-J treatment of U87 MG brain cancer cells. In addition, we performed Western blotting with apoptosis-related proteins to understand mechanisms. Our study shows that caspase-3, caspase-7, and PARP are upregulated and may activate complex intracellular apoptotic cascades, ultimately promoting apoptosis. We found that NBP-J can increase levels of apoptosis-related proteins, dependent on plasma treatment time. The location of p-Akt expression was also determined by immunofluorescence staining. Both cytoplasmic and nuclear p-Akt decreased significantly after plasma treatment. Moreover, reactive species generated by NBP-J can induce apoptosis by MAPK signaling, including downregulation of ERK1/2 and JNK, and upregulation of p38 MAPK.

Finally, generated RONS entered cells and blocked the PI3K/Akt pathway that upregulates p38, down-regulating the JNK and ERK1/2 signal. RONS can also increase brain cancer cell apoptosis by activating p53, caspase-3, caspase-7, and PARP. Our findings give a fundamental basis for the improvement of a clinically fitting device to be directly applied to the brain. However, for our further simple protocol for in vivo study, it will be semi-open surgical. It means we can open the skin of the head and then make a hole using a syringe, then treat it with a plasma jet through the hole. Stereotaxic device using a Hamilton syringe will be used to inject the cells. Stereotaxic coordinates will be selected to be 1 mm anterior and 2 mm lateral to the bregma and 3 mm deep from the dura. After injecting the cancer cells, they will be treated directly.

We will use soft jet plasma to treat the brain tissue at approximately 5 mm distance from the tip of the plasma discharge and the treatment will last for 30 s each time and we will make some gap time. That is our vision of the tumor modeling procedure for this in vivo study as a semi-open surgical method. We have established this animal model method and successfully performed it so far.

## 5. Conclusions

In this study, we found that NBP-J treatment can be used to treat brain cancer cells therapeutically. By comparing the apoptotic impacts in cancer cells with those in astrocytes, we demonstrated that NBP-J can specifically stimulate apoptosis in brain cancer cells through the formation of reactive species. In general, NBP-J treatment induced morphological changes, cell death, and cell cycle arrest, and ultimately increased apoptotic protein expression in the U87 MG cancer cell line. Our results may provide basic information about the mechanism relevant to ROS, which leads to cell death. NBP-J generated different reactions in cancer and non-cancer cells, suggesting that it may be a successful new therapy. Furthermore, NBP-J has been operated at room temperature in the open air, particularly notable by plasma operating parameters. In summary, this therapy may be made selective to cancerous cells over normal cells in a clinically suitable device.

## Data Availability

Not applicable.
